# Adult Medulloblastoma Demographic, Tumor and Treatment Impact since 2006: A Canadian University Experience

**DOI:** 10.3390/curroncol28040271

**Published:** 2021-08-14

**Authors:** Maria Camila Quinones, Karl Bélanger, Émilie Lemieux Blanchard, Bernard Lemieux, Jean-Paul Bahary, Laura G. Masucci, David Roberge, Cynthia Menard, Carole Lambert, France Berthelet, Robert Moumdjian, Marie Florescu

**Affiliations:** 1 CHUM Research Centre (CrCHUM), Montreal, QC H2X 0A9, Canada; 2 CHUM Research Centre (CrCHUM), Department of Hematology and Oncology, Centre Hospitalier de l’Université de Montréal (CHUM), Montreal, QC H2X 3E4, Canada; karl.belanger.chum@ssss.gouv.qc.ca (K.B.); emilie.lemieux-blanchard.chum@ssss.gouv.qc.ca (É.L.B.); bernard.lemieux.chum@ssss.gouv.qc.ca (B.L.); jean-paul.bahary.chum@ssss.gouv.qc.ca (J.-P.B.); g.laura.masucci.chum@ssss.gouv.qc.ca (L.G.M.); david.roberge.chum@ssss.gouv.qc.ca (D.R.); cynthia.menard@umontreal.ca (C.M.); carole.lambert.chum@ssss.gouv.qc.ca (C.L.); france.berthelet@umontreal.ca (F.B.); robert.a.moumdjian.med@ssss.gouv.qc.ca (R.M.); marie.florescu.med@ssss.gouv.qc.ca (M.F.)

**Keywords:** medulloblastoma, adult medulloblastoma, chemotherapy, prognostic factors

## Abstract

Medulloblastoma is an aggressive primary brain tumor that is extremely rare in adults; therefore, prospective studies are limited. We reviewed the information of all MB patients treated at the CHUM between 2006 and 2017. We divided our cohort by age and further divided adult patients (53%) in two groups, those diagnosed between 2006–2012 and 2013–2017. In our adult population, median follow up was 26 months and SHH-activated MB comprised 39% of tumors. Adult 5yOS was 80% and first-line therapy led to a 5yPFS of 77%. The absence of radiosensitizing chemotherapy (100% vs. 50%; *p* = 0.033) negatively influenced 5yPFS. 96% of adult patients received radiotherapy and 48% of them received concomitant radiosensitizing chemotherapy. Complete surgical resection was performed on 85% of adults, but the extent of resection did not have a discernable impact on survival and did not change with time. Adjuvant chemotherapy did not clearly affect prognosis (5yOS 80% vs. 67%, *p* = 0.155; 5yPFS 78% vs. 67%, *p* = 0.114). From 2006–2012, the most common chemotherapy regimen (69%) was Cisplatinum, Lomustine and Vincristine, which was replaced in 2013 by Cisplatinum, Etoposide and Cyclophosphamide (77%) with a trend for worse survival. Nine patients recurred and seven of these (78%) were treated with palliative chemotherapy. In conclusion, we did not identify prognostic demographic or tumor factors in our adult MB population. The presence of radiosensitizing chemotherapy was associated with a more favorable PFS. Cisplatinum, Lomustine and Vincristine regimen might be a better adjuvant chemotherapy regimen.

## 1. Introduction

Medulloblastoma (MB) is the most common malignant brain tumor of childhood, accounting for nearly 20% of all primary central nervous system (CNS) tumours amongst patients younger than 19 years of age. It is extremely rare in adults, representing less than 1% of intracranial tumors in this population [1,2]. The incidence rate of MB in adults is 0.5 cases/million, making prospective studies in this age group remarkably limited and controlled randomised trials non-existent [3]. Consequently, the available literature largely focuses on paediatric MB and controversy remains regarding adult prognostic factors and standard-of-care treatment.

Literature suggests that childhood and adult MB differ clinically and phenotypically [4,5,6]. Thus, evidence indicates that the different factors that affect patients’ prognosis vary according to their age. Notably, adults tend to have numerous comorbidities when compared with children. These differences have brought the scientific community to question that adults generally receive the same therapies developed for children. In this study, we aimed to determine the survival of the adult MB patients treated at our centre, as well as to evaluate their prognostic factors and treatment efficacy.

## 2. Materials and Methods

### 2.1. Data Source and Patient Selection

We conducted a retrospective study of all MB patients treated at the *Centre hospitalier de l’Université de Montréal* (CHUM) using our tumor registry and electronic medical records (EMR). First, through our tumour registry, which actively collects data from patients seen in the cancer center, we identified patients with MB diagnosed between 2006 and 2017. From this database, we extracted age at MB diagnosis, sex, family history of central nervous system (CNS) tumors, tumour histology and molecular markers, progression dates, date of last contact, date of death and treatment regimens received. We then completed this information using our general and oncology EMRs.

The study protocol was submitted and approved by the Research Ethics Committee of the CHUM Research Centre (CRCHUM).

Of all 50 patients identified by our tumour registry, 49 were included in the study. One was excluded from the analysis as he only made one visit to our emergency department for a reason unrelated to his MB diagnosis. All patients identified had histologically confirmed MB. The median follow-up for adult patients was 26 months, with a range of 3 months to 7 years.

Of the patients identified, 23 were children and 26 were adults. Children were typically only seen in our cancer centre for radiotherapy and thus had more limited follow-up information.

### 2.2. Patient Distribution

**Figure 1:**
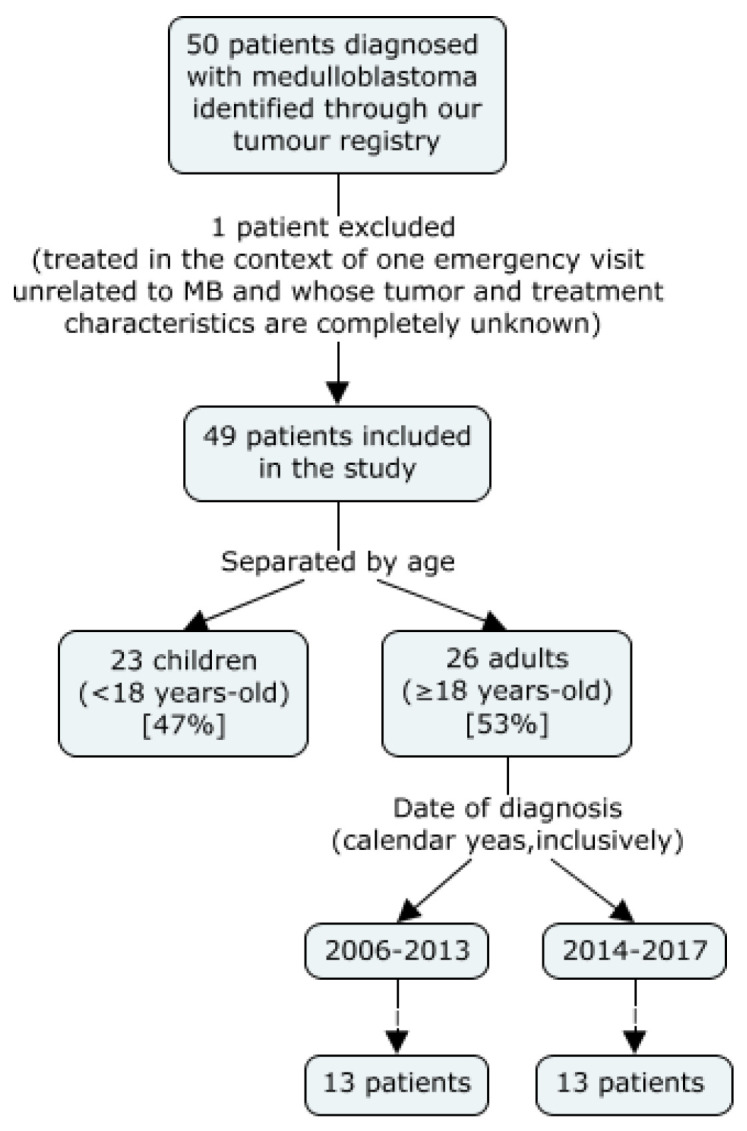
Patient Selection and Distribution.

As observed in Figure 1, we divided the adults (18 years of age and older) in two sub-groups, those diagnosed between 2006 and 2012 and those diagnosed between 2013 and 2017. The cut-off of 2012 was based on a change in the adjuvant chemotherapy regimen used. Before 2013, a Cisplatinum and Lomustine-based regimen was most frequently prescribed; whereas after this date, a Cisplatinum and Etoposide-based regimen predominated. This change took place as evidence suggesting an advantage of the new topoisomerase II inhibitor (TOP2i)-based regimen compared to the old nitrosourea-based one became more available. Notably, a study conducted by Silvani et al. and published at the end of 2011 concluded that patients exposed to this new regimen experienced “considerably lower chemotherapy-related toxicity, compared with that experienced by patients who received other treatments” [3].

### 2.3. Outcomes

The primary outcome of this study was the demographic, tumor and treatment characteristics specific to our population and their impact on overall and progress-free survival. We compared these findings to the paediatric and adult populations that have previously been described and aimed to recommend a first line treatment regimen for adults.

### 2.4. Statistical Analysis

Overall survival (OS) was defined as the time from diagnosis until death by any cause. Progress-free survival (PFS) was defined as the time from diagnosis until the first recurrence, or death if no recurrence had been confirmed previously. The date of diagnosis was determined as the date of tumour extraction. Recurrence was defined by a significant increase in measurable disease by imaging studies, or the development of a new tumour locus, including local or distant metastasis. The Kaplan–Meier method was used to assess OS and PFS.

Patient characteristics (age and gender), tumour characteristics (histopathology, molecular subgroup and extent of disease), and treatment-related factors (extent of surgical resection, radiotherapy use with and without concomitant chemotherapy, adjuvant chemotherapy use and its different agents) were analyzed as potential prognostic factors using the univariate log-rank method. A *p*-value < 0.05 was considered statistically significant. Due to the limited population size of our study, we were unable to define prognostic variables by a multivariate analysis.

The IBM SPSS Statistics software (IBM Corp. Released 2017. IBM SPSS Statistics for Windows, Version 25.0. IBM Corp, Armonk, NY, USA) was used for statistical analysis.

## 3. Results

### 3.1. Patient and Tumour Characteristics

Of our 49 patients, 23 were children (47%) and 26 were adults (53%). Two patients were younger than 3 years of age (4%) and nine were aged between 40 and 60 (18% of all patients and 35% of adult patients). There were no patients older than 60 years. The median age of our adult cohort was 29 (18 to 53 years-old) and the median follow-up was 26 months. This is remarkably similar to some of the largest multicentre studies available [7,8,9,10,11]. The demographic, tumour and treatment characteristics of the population in our study are summarized in Table 1.

In a preliminary analysis, we compared survival for patients between 18 and 39 years of age to those between 40 to 60 years of age. Because there was no significant difference between the two groups, we decided to study all adults as one group.

Contrary to the observed positive prognostic value of female sex in the paediatric population, the effect of sex on the progression of adult disease remains controversial [12,13,14]. Our cohort was composed mainly of men (73%), which is consistent with available literature [1,3,5,6,8,9,10,12,15,16,17], and sex had no discernable impact on OS or PFS.

In children, histology clearly is of prognostic significance. In adults, there is consensus that LCAMB is associated with worse outcomes, but there is contradictory data regarding CMB and DNMB, which are the most frequent histologic variants [5,8,15,18,19,20]. Despite being more common in adults than in children, DNMB’s prognostic significance has not been clearly established in the adult population [5,11,21]. In our study, CMB and DNMB were the most frequent histological variants (42% and 46% respectively) and histopathology did not significantly influence OS or PFS. However, differences in therapy may have influenced this result because patients with CMB more often received RT with concomitant chemotherapy and a nitrosourea-based adjuvant chemotherapy regimen, compared with patients with DNMB, which more often received RT alone and a TPO2i-based adjuvant chemotherapy regimen.

During the past decade, increasing emphasis has been placed on the impact of molecular subgroups on patient prognosis. In most available adult studies, a predominance of non-WNT/non-SHH tumours (~60% and almost exclusively Group 4), followed by SHH-activated tumours (~30%), and very rarely WNT-activated tumours (~10%) has been reported [6,9,22]. Contrarily, in our study, there was a similar prevalence of non-WNT/non-SHH and SHH-MB (42% vs. 39%) and there were no patients with WNT-MB amongst adults with available molecular characteristics (81%). Moreover, in our cohort, molecular subgroups did not have a significant impact on patient outcome, contrary to previous studies where SHH-MB has been linked to better survival [6,22].

The demographic, tumour and summarized treatment characteristics for adult and paediatric patients is presented in Table 1.

### 3.2. Treatment Modalities

In our cohort, 22 adult patients (85%) had a complete macroscopic tumour resection. All but one patient received radiotherapy (RT). The one patient who did not receive RT had M0-stage disease and decided to go into palliative care after complete surgical resection of his tumour. A total of 13 patients (50%) received RT alone and 12 patients (46%) received RT with concomitant chemotherapy (RTCC). The radiosensitizing chemotherapy agent used in all patients was Vincristine.

Adjuvant (maintenance) chemotherapy was given to 21 adult patients (81%). Nine patients (43%) received a Cisplatinum and Lomustine-based regimen, ten patients (48%) received a Cisplatinum and Etoposide-based regimen and the remaining two (10%) received a different regimen (Table 2).

Only three patients did not receive chemotherapy at any point in time. One patient chose to receive no adjuvant therapy, one moved out of the country before completing RT, and one patient was prescribed RT alone as his adjuvant therapy. The latter had disease recurrence after five years and was treated with palliative chemotherapy before dying seven years after his diagnosis.

Beginning in 2013, the most prescribed adjuvant chemotherapy regimen at our centre drastically drifted from a nitrosourea-based treatment to a TOP2i-based one. Because of this, we decided to compare the outcome of patients diagnosed between 2006 and 2012 with those diagnosed between 2013 and 2017. Each group had 13 patients, 11 (85%) of which had a complete macroscopic tumour resection, and two (15%) had a partial resection. All patients diagnosed before 2013 received RT and most of them (85%) received RTCC. Amongst those diagnosed after 2013, all patients but one received RT, and most of those who did (85%) received RT without concurrent chemotherapy.

A *p*-value to compare both groups based on baseline characteristics could not be calculated as χ^2^ cannot be used (most expected frequencies are less than five).

Amongst the three adult patients who had disease progression, two received salvage chemotherapy with temozolomide and one underwent surgery and an allogenic stem cell transplant.

### 3.3. Survival

**Figure 2:**
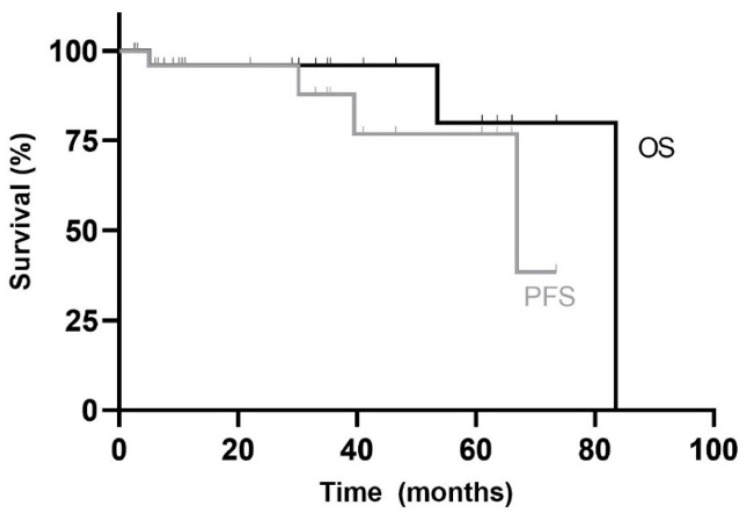
Kaplan–Meier overall survival (OS) and progress-free survival (PFS) curves for adult patients in the study. Median follow-up duration was 26 months (range: 3 months to 7 years).

The median OS of adults in our cohort was 83.5 months with an OS of 80% at five years. First line therapy had a median PFS of 67 months with a 5yPFS of 77% (Figure 2).

**Figure 3:**
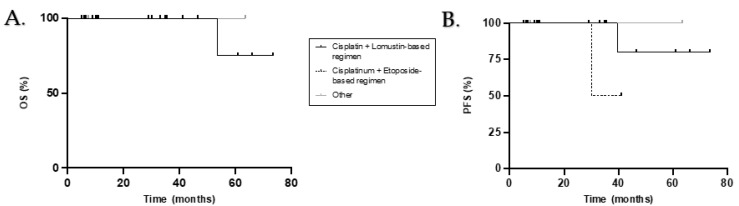
Kaplan–Meier overall survival (**A**) and progress-free survival (**B**) curves for adult patients who received adjuvant chemotherapy according to treatment regimen.

Table 3 summarizes the OS and PFS rates according to the different demographic, tumour and treatment characteristics. Sex, histopathology, molecular subgroup, extent of disease, quality of resection and presence or type of adjuvant chemotherapy were not significantly associated with OS or PFS (Figure 3). Only the type of RT received had prognostic value. In fact, patients who received chemotherapy alongside RT (RTCC) had a significantly better PFS compared with patients who received RT alone (100% vs. 50% at 5 years; *p* = 0.033) and had a tendency for a better OS (100% vs. 50% at 5 years; *p* = 0.157). A trend for better OS and PFS was observed in patients who received adjuvant chemotherapy compared to those who did not (*p* = 0.155 and *p* = 0.114 respectively).

**Figure 4:**
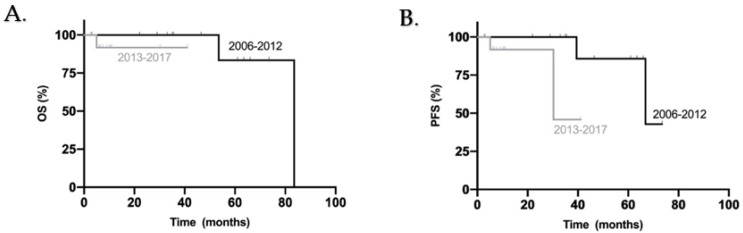
Kaplan–Meier overall survival (**A**) and progress-free survival (**B**) curves for all adult patients included in this study according to year of diagnosis.

When comparing both groups, we highlight a tendency for better PFS in patients diagnosed from 2006 to 2012 (*p* = 0.087) and no significant difference for OS between the two groups (*p* = 0.317) (Figure 4).

## 4. Discussion

Our study comprises the most recent adult MB patient series in North America and is the only series consisting of primarily French-Canadian patients. All patients were followed at the main reference centre for adult MB in the province of Quebec. Given the rarity of MB in the adult population, this review stands as one of the biggest ones of its kind in current literature.

The characteristics of the children treated at the CHUM was included in Table 1 for reference and comparison to the paediatric population that has been described in literature, as well as to the adult population specific to this study. Children were not included in our discussion as the scope of this study is limited to adults. Furthermore, the information on the treatment regimen used in children was limited as they were typically seen at our centre for radiotherapy only.

### 4.1. Survival

Adult MB patients in the present study had better OS and PFS than typically described in the available literature. In multiple retrospective adult MB series analysing 13 to 206 patients, the 5yOS ranged between 40 and 84% and the 5yPFS between 32 and 63% [1,8,9]. In our cohort, the 5yOS was 80% and the 5yPFS was 77%.

### 4.2. Impact of Demographic and Tumour Factors

In our study, age, sex and histological subtypes had no impact on the prognosis of adult MB patients. Therefore, we believe that our outstanding OS and PFS is due, in part, to the high prevalence of SHH-activated tumours in our cohort when compared to current literature.

Advanced disease at diagnosis is a recognized prognostic factor in children, but it is of debatable relevance in adults [19]. Several adult studies have observed that there is no correlation between M-stage at diagnosis and patient outcome [11,12,19]. In our study, this was difficult to evaluate as information was unavailable for 42% of patients; CSF analysis was lacking, either because it was done at another centre and the results were not reported, or because it was never performed. Following a review of their imaging studies, it was determined that no patient in the unavailable M-stage category had macroscopic metastasis (M2–M4) and we can now assume, based on our excellent OS and PFS, that CSF was negative for most of them. However, as obtaining CSF in all patients is Level I A evidence, we are committed to improving this aspect of patient care [23].

### 4.3. Role of the Different Treatment Variables

Adults continue to be treated with regimens extrapolated from paediatric protocols despite the differences between adult and paediatric MB since the rareness of the tumour hinders prospective studies and randomized control trials. Maximal safe surgical resection aiming for gross total tumour extraction and RT with CSI are recognized to be standard-of-care for adults [23,24,25,26,27]. However, the advantage of chemotherapy during or after RT remains controversial [3,12,23,27,28].

In our study, we demonstrated that 38% of adult patients received a full treatment including RT, concomitant chemotherapy (RTCC) and adjuvant chemotherapy in spite of their age, comorbidities and disease extent. All patients but one received RT and nearly half of these patients received concomitant chemotherapy (Vincristine). Patients who received RT alone had significantly worse PFS (5yPFS 50% vs. 100%, *p* = 0.033) when compared with patients who received RTCC. The same trend was observed with regards to OS (5yOS 50% vs. 100%, *p* = 0.157).

We did not demonstrate a significant advantage in the administration of maintenance chemotherapy, but we observed a tendency for better PFS and OS in patients who did receive it (5yOS 80% vs. 67%, *p* = 0.155; 5yPFS 78% vs. 67%, *p* = 0.114). This correlates with the many available studies and guidelines that suggest its use in all adult patients regardless of risk stratification [17,19,23,29,30,31]. Patients who received RTCC followed by maintenance chemotherapy had statistically better OS and PFS when compared with patients who received RT alone followed by maintenance chemotherapy, regardless of chemotherapy agent (*p* = 0.046 and *p* = 0.023 respectively). We did not demonstrate a clear advantage of a Cisplatinum and Lomustine based regimen when compared with Cisplatinum and Etoposide but observed a tendency for a better PFS in patients treated with the former agents (*p* = 0.087).

### 4.4. Limitations

The retrospective nature of our review and its small sample size comprise its major limitations. However, given the rarity of MB in the adult population, this study remains one of the most important of its kind in current literature. Furthermore, this review revealed a lack of complete workup in patients prior to treatment (i.e., CSF analysis). As explained above, this made it more difficult to precisely classify patients according to their stage at diagnosis (i.e., M0 vs. M1 disease). As there is high level evidence supporting complete workup in patients prior to treatment, we are committed to improving this aspect of patient care [23]. Still, we have demonstrated an excellent OS and PFS in our adult population.

## 5. Conclusions

Our adult MB population has an outstanding OS and PFS when compared to children and adults in most available studies, despite most patients not receiving a full treatment regimen when compared to paediatric standards (surgical resection, RT, RTCC and adjuvant chemotherapy). The demographic and tumor characteristics did not have a discernable impact on prognosis. Radiotherapy with concurrent chemotherapy (Vincristine) had a significantly favourable impact on PFS and a tendency for better OS. We observed a trend for a more favourable outcome in patients who received adjuvant chemotherapy, especially with a Cisplatinum, Lomustine and Vincristine regimen. Prospective studies are required to further evaluate the roles of RTCC and adjuvant chemotherapy.

## Figures and Tables

**Table 1:** Patient, Tumor and Summarized Treatment Characteristics.

		All Participants N = 49 (%)	Adults N = 26 (%)	Children N = 23 (%)
**Demographic Characteristics**				
Median Age in Years (range)		19 (0–53)	29 (18–53)	7 (0–16)
Sex	Male	36 (73)	19 (73)	17 (74)
	Female	13 (27)	7 (27)	6 (26)
Family history of CNS cancer	Yes	-	-	-
	No	15 (31)	15 (58)	-
	Unknown	34 (69)	11 (42)	23 (100)
Comorbidities	Yes	4 (8)	4 (15)	-
	No	45 (92)	22 (85)	23 (100)
**Tumor Characteristics**				
Histopathology	Classic (CMB)	26 (53)	11 (42)	15 (65)
	Desmoplastic/nodular (DNMB)	16 (33)	12 (46)	4 (17)
	Large cell/anaplastic (LCAMB)	7 (14)	3 (12)	4 (17)
Molecular Subgroup *	SHH-activated (SHH-MB)	13 (27)	10 (39)	3 (13)
	SHH- activated: TP53-mutant	1 (2)	-	1 (4)
	SHH activated: TP53-wildtype	12 (25)	10 (39)	2 (9)
	Non-WNT/non-SHH **	25 (51)	11 (42)	14 (61)
	Unknown	11 (22)	5 (19)	6 (26)
Stage at Diagnosis ***	M0	30 (61)	14 (54)	16 (70)
	M2	3 (6)	1 (4)	2 (9)
	M3	3 (6)	-	3 (13)
	Unknown	13 (27)	11 (42)	2 (9)
Cerebral Spinal Fluid (CSF) at Diagnosis	Positive	1 (2)	1 (4)	-
	Negative	11 (22)	6 (23)	5 (22)
	Unknown	37 (76)	19 (73)	18 (78)
Tumor Location	Cerebellum	40 (82)	23 (88)	17 (74)
	Brainstem	9 (18)	3 (12)	6 (26)
**Treatment Characteristics**				
Surgical macroscopic resection	Complete	43 (88)	22 (85)	21 (91)
	Partial	6 (12)	4 (15)	2 (9)
Radiotherapy	None	1 (2)	1 (4)	-
	Alone	25 (51)	13 (50)	12 (52)
	With concomitant chemotherapy	23 (47)	12 (46)	11 (48)
Adjuvant Chemotherapy	Yes	22 (45)	21 (81)	1 (4)
	No	5 (10)	5 (19)	-
	Unknown ****	22 (45)	-	22 (96)

Percentages might not add up to 100 because of rounding. * There were no patients with a WNT-activated pathway tumour (WNT-MB). ** Equivalent to Subgroups 3 and 4. *** Information according to the medical record. There were no patients with Stage M1 or M4 disease at diagnosis. **** Information not available due to patients being followed at a paediatric centre (only intended regimen was available in our EMRs).

**Table 2:** Adult Baseline Characteristics and Treatment Regimen Based on Year of Diagnosis (Inclusively).

		All Adults N = 26 (%)	2006–2012 N = 13 (%)	2013–2017 N = 13 (%)
**Demographic and Tumour Characteristics**				
Median Age in Years (range)		29 (18–53)	28 (20–53)	31 (18–51)
Sex	Male	19 (73)	10 (77)	9 (69)
	Female	7 (27)	3 (23)	4 (31)
Histopathology	Classic (CMB)	11 (42)	8 (61.5)	3 (23.5)
	Desmoplastic/nodular (DNMB)	12 (46)	4 (31)	8 (61.5)
	Large cell/anaplastic (LCAMB)	3 (12)	1 (7.5)	2 (15)
Molecular Subgroup	SHH-activated *	10 (39)	-	10 (77)
	Non-WNT/non-SHH	11 (42)	9 (69)	2 (15)
	Unknown	5 (19)	4 (31)	1 (7.5)
Stage at Diagnosis	M0	14 (54)	5 (38.5)	9 (69)
	M2	1 (4)	-	1 (7.5)
	Unknown	11 (42)	8 (61.5)	3 (23)
**Treatment Variables**				
Surgical Macroscopic resection	Complete	22 (85)	11 (85)	11 (85)
	Partial	4 (15)	2 (15)	2 (15)
Radiotherapy	None	1 (4)	-	1 (7.5)
	Alone	13 (50)	2 (15)	11 (85)
	With induction/concomitant chemotherapy	12 (46)	11 (85)	1 (7.5)
First Intention Maintenance Chemotherapy	**None**	5 (19)	3 (23.5)	2 (15)
	**Cisplatinum + Lomustine-based regimen**	9 (43)	9 (69)	-
	Cisplatinum + Lomustine **	1 (5)	1 (7.5)	-
	Cisplatinum + Lomustine + Vincristine	8 (38)	8 (61.5)	-
	**Cisplatinum + Etoposide-based regimen**	10 (48)	-	10 (76.5)
	Cisplatinum + Etoposide	1 (5)	-	1 (7.5)
	Cisplatinum + Etoposide + Cyclophosphamide	9 (43)	-	9 (69)
	**Other**	2 (10)	1 (7.5)	1 (7.5)
	Temozolomide	1 (5)	1 (7.5)	-
	Vincristine + Cisplatinum + Cyclophosphamide alternating with Premetrexed and Gemcitabine	1 (5)	-	1 (7.5)

Group A: diagnosed between 2006 and 2012. Group B: diagnosed between 2013 and 2017. Percentages might not add up to 100 because of rounding. * All adult patients with a SHH-activated pathway tumour had a TP53-wildtype variant. ** This patient did not receive vincristine because of non-resolving neuropathies after RT.

**Table 3:** Univariate Analysis on the Impact of Patient, Tumour and Treatment Characteristics on Overall and Progress-Free Survival in Adult Patients.

		No. of Pts *n* = 26	5yOS (%)	*p*-Value	5yPFS (%)	*p*-Value
**Demographic and Tumour Characteristics**						
Sex	Male	19	75	0.515	75	0.642
	Female	7	83.3		83.3	
Histopathology	Classic (CMB)	11	100	0.368	100	0.162
	Desmoplastic/nodular (DNMB)	12	61.1		68.8	
	Large cell/anaplastic (LCAMB)	3	100		100	
Molecular Subgroup	SHH-activated	10	NR	0.412	NR	0.148
	Non-WNT/non-SHH	11	80		80	
	Unknown	5	NR		NR	
Stage at Diagnosis	M0	14	92.3	0.889	73.8	0.496
	M2	1	NR		NR	
	Unknown	11	80		80	
**Treatment Variables**						
Surgical Macroscopic Resection	Complete	22	79.2	0.655	74.8	0.527
	Partial	4	NR		NR	
Radiotherapy	Alone	13	50	0.157	50	0.033
	With induction/concomitant chemotherapy	12	100		100	
Adjuvant Chemotherapy	Yes	21	80	0.155	77.9	0.114
	No	5	66.7		66.7	
Type of Adjuvant Chemotherapy (First Intention)	Cisplatinum + Lomustine-based regimen	9	75	-	80	0.410
	Cisplatinum + Etoposide-based regimen	10	NR		NR	
	Other	2	NR		NR	

NR: not reached.

## Data Availability

The data presented in this study are available on request from the corresponding author. The data are not publicly available due to privacy and ethical reasons.
